# Atrazine in sub-acute exposure results in sperm DNA disintegrity and nuclear immaturity in rats

**Published:** 2012

**Authors:** Sajad Feyzi-Dehkhargani, Rasoul Shahrooz, Hassan Malekinejad, Rajab-Ali Sadrkhanloo

**Affiliations:** 1 *Department of Comparative Histology & Embryology, Faculty of Veterinary Medicine, Urmia University, Urmia, Iran; *; 2 *Department of Pharmacology and Toxicology, Faculty of Veterinary Medicine, Urmia University, Urmia, Iran.*

**Keywords:** Atrazine, Cytoplasmic carbohydrate and lipid Spermatogenesis, DNA damage, Endocrine function

## Abstract

This study was designed to evaluate the detrimental effect of atrazine (ATR) on germinal epitheliums (GE) cytoplasmic carbohydrate (CH) and unsaturated fatty acids (UFA) ratio and to clarify the effect of ATR on serum levels of FSH, LH, testosterone and inhibin-B (INH-B). The impact of ATR exposure on total antioxidant capacity (TAC), sperm DNA packing and integrity were also investigated. Seventy two Wistar rats were used. The rats in control group received vehicle and the animals in test groups received 100, 200 and 300 mg kg^-1^ BW of ATR orally on daily bases for 12, 24 and 48 days. In ATR-received groups the spermatogenesis cell were presented with dense reactive sites for lipidophilic staining associated with faint cytoplasmic CH accumulation. Dissociated germinal epithelium, negative tubular and repopulation indexes were manifested. The serum levels of testosterone, FSH, LH and INH-B decreased by 85% after 48 days exposure to high dose of ATR. TAC was reduced in a time- and dose-dependent manner. The sperm DNA damage was marked in animals which exposed to high dose of ATR (72.50 ± 2.25%) and the percentage of nuclear immature sperm increased up to 83.40 ± 0.89%. In conclusion, ATR not only induced its detrimental effect on the endocrine function of the testes and pituitary gland but also affected the cytoplasmic CH ratio and consequently leads to inadequate energy supplement in spermatogenesis cells. Therefore the imbalanced oxidative stress occurs in testicular tissue, which in turn enhances the sperm DNA disintegrity and nuclear immaturity.

## Introduction

Atrazine (2-chloro-4-ethylamino-6-isopropylamino-s-triazine; ATR) is a chloro-s-trazine herbicide, which is used extensively worldwide for broadleaf and grassy weed control in corn, sorghum, sugarcane, cotton, and pineapple crops and landscape vegetation.^1^ ATR and its metabolites (deethylatrazin) are highly persistent in water and are mostly found in soil especially in farming seasons.^2^ It has been reported that in the United States approximately 75 million pounds of ATR are used annually, which is more than other herbicides. ATR remains on corns and/or sugarcane for a long time after one time usage in farming season.^3^ According to previous reports there is a positive correlation between testicular degeneration and exposure to toxic compounds, especially those which are capable to disrupt endocrine functions.^4^^-^^6^ During the last decade there was an increasing focus on clarifying the mechanism(s) of toxicities of environmental toxicants, which are able to cause alterations in the reproductive system. There are reports indicating the ATR-induced toxicities on testicular tissue.^5^ The effect of ATR however on germinal cells cytoplasmic carbohydrate (CH) and unsaturated fatty acids (UFA) ratio has not been fully addressed yet. Moreover, ATR-induced detrimental impacts on spermatogenesis cells have been remained as a controversial matter. The first aim of the current study was to evaluate the cytoplasmic CH and UFA alterations in germinal epithelium and to illustrate possible changes in seminiferous tubules (STs).

ATR like other herbicides induces endocrine disruption and consequently interferes with various hormones physiological functions.^7^ The indirect influence of ATR on the pituitary gonadal axis of the male offspring was studied by the treatment of dams during pregnancy and/or both during pregnancy and lactation.^8^^-^^12^ In the male offspring a slower maturation of the gonadotrophic system due to chronic exposure to ATR was observed.^13^^-^^15^ Therefore the second aim of present study was to evaluate any alterations in serum levels of testosterone, inhibin-B, luteinizing hormone (LH) and follicle stimulating hormone (FSH). It is well understood that, the lowered semen quality correlates positively with amount of reactive oxygen species (ROS) in reproductive system.^16^^-^^19^ On the other hand like other herbicides such as glyophosate, imdoclopride, ATR is capable to induce oxidative stress in entire body.^6^ Thus the last aim of the current research was to evaluate the relationship between ATR-induced oxidative stress and sperm DNA intensity and packing.

## Materials and Methods

**Animals. **Seventy two mature male Wistar rats, 8 weeks old and 200 ± 15 g weight, were used. The rats were obtained from the Animal House of the Faculty of Veterinary Medicine, Urmia University, Iran. They were allowed to acclimatize in an environmentally controlled room (temperature, 20-23 °C, and 12h light/12h dark). Food and water were given *ad libitum*. In this study all experiments which conducted on animals were in accordance with the guidance of ethical committee for research on laboratory animals of Urmia University.

**Experimental design. **The animals were assigned into 12 groups (n = 6) as control and test groups. All animals prior to beginning of the experiments and after the last step of the treatment were weighed to evaluate any changes in body weight gain (BWG). Animals in control groups received the corn oil (0.2 mL per day) and the rats in the test groups were administered ATR at dose levels of 100 mg kg^-1^ BW, (low dose: LD), 200 mg kg^-1^ BW, (medium dose: MD), and 300 mg kg^-1^ BW, (high dose: HD) orally once a day for 48 days. The rats were sampled on days 12, 24, 48 after dosing.

**Histopathological analysis**. One half of the testes specimens were fixed in 10% formalin for histological examinations and subsequently embedded in paraffin. Sections (5-6 µm thick) were stained with Iron-Weigert (Pajohesh Asia, Iran) for the detection of germinal cells nucleuses in the testes. For the quantification of germinal cells a special morphometrical lens–device (100µm, Olympus Co., Germany) was used. 

**Histochemical study.** Another half of the specimens were freshly cut by frozen section to analyze the testicular germinal epithelium carbohydrate ratio by using the periodic acid schiff (PAS) special staining. Furthermore the Sudan Black B (SB) staining was performed to evaluate the rate of lipid foci supplement in germinal epithelium. 

**Sperm abnormality, nuclear maturity and DNA integrity. **After the collection of epididymal sperm, the sperm count was conducted using the hemocytometer.^6^ The aniline blue staining was performed to evaluate the number of sperms with nucleus maturity. The sperms with light stained nucleuses were considered as nuclear matured cells. The acridine orange staining technique (special staining for DNA double-strand integrity) was conducted on smeared specimens using epi-fluorescent microscope (Olympus, Germany), as well. Eosin-negrosin staining was conducted to evaluate the morphologically immature (MI) sperms. The sperms with cytoplasmic residuals were considered as MI sperms. 

**Hormonal analysis. **After days 12, 24 and 48 the blood samples from corresponding animals were collected directly from the heart and the serum samples separated (Hittech, EBA III-Japan). The collected serum samples were subjected to hormonal analysis. The serum level of testosterone, INH-B, FSH and LH were measured using the radioimmunoassay method. The intra-assay and inter-assay coefficients variances for testosterone, INH-B, FSH and LH was found 4.8, 4.3, 3.7and 4.1 (for 10 times) and 9.9, 8.4, 8.3 and 7.9 (for 10 times), respectively.

**Assessment of the total antioxidant capacity (TAC). **To determine the effect of ATR on oxidative stress system total antioxidant capacity were measured. The assessment carried out based on ferric reduction antioxidant power (FRAP) assay.^20^ Briefly, at low pH which was provided using acetate buffer (300 mM, pH 3.6), reduction of Fe(III)-TPTZ complex to the ferrous form produces an intensive blue color that could be measured at 593 nm. The intensity of the complex following adding the appropriate volume of the serum to reducible solution of Fe (III)-TPTZ is directly related to total reducing power of the electron donating antioxidant. Aqueous solution of Fe (II) (FeSO4 - 7H2O0) and appropriate concentration of freshly prepared ascorbic acid were used as blank and standard solutions, respectively.

**Statistical analyses. **The statistical analyses were performed on all numerical data by using two-way ANOVA and using SPSS software version 13.0. All values were expressed as the mean ± SD. *P* < 0.05 was considered to be statistically significant. For comparing the graded degree of histological findings between groups, the Kruskal-Wallis test was used.

## Results

**Clinical observations. **The animals in test groups manifested with reduced total BWG. The testicular weight determination showed that following ATR administration the total testicular weight gain decreased in all tested animals in comparison to the control group (Table 1).

**Fig. 1:**
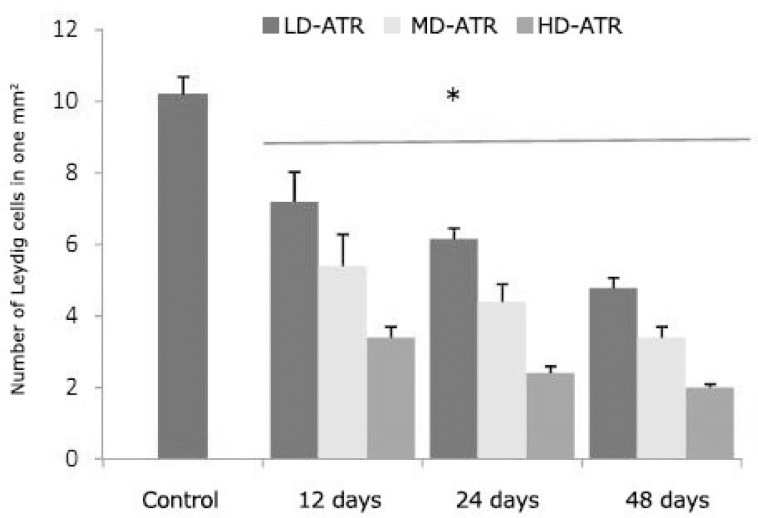
Average of the Leydig cells distribution in one mm^2 ^of the interstitial connective tissue; the star is indicating significant differences (*P* ≤ 0.05) between all test groups with each other and with control group. All data are presented as mean ± SD.

**Fig. 2:**
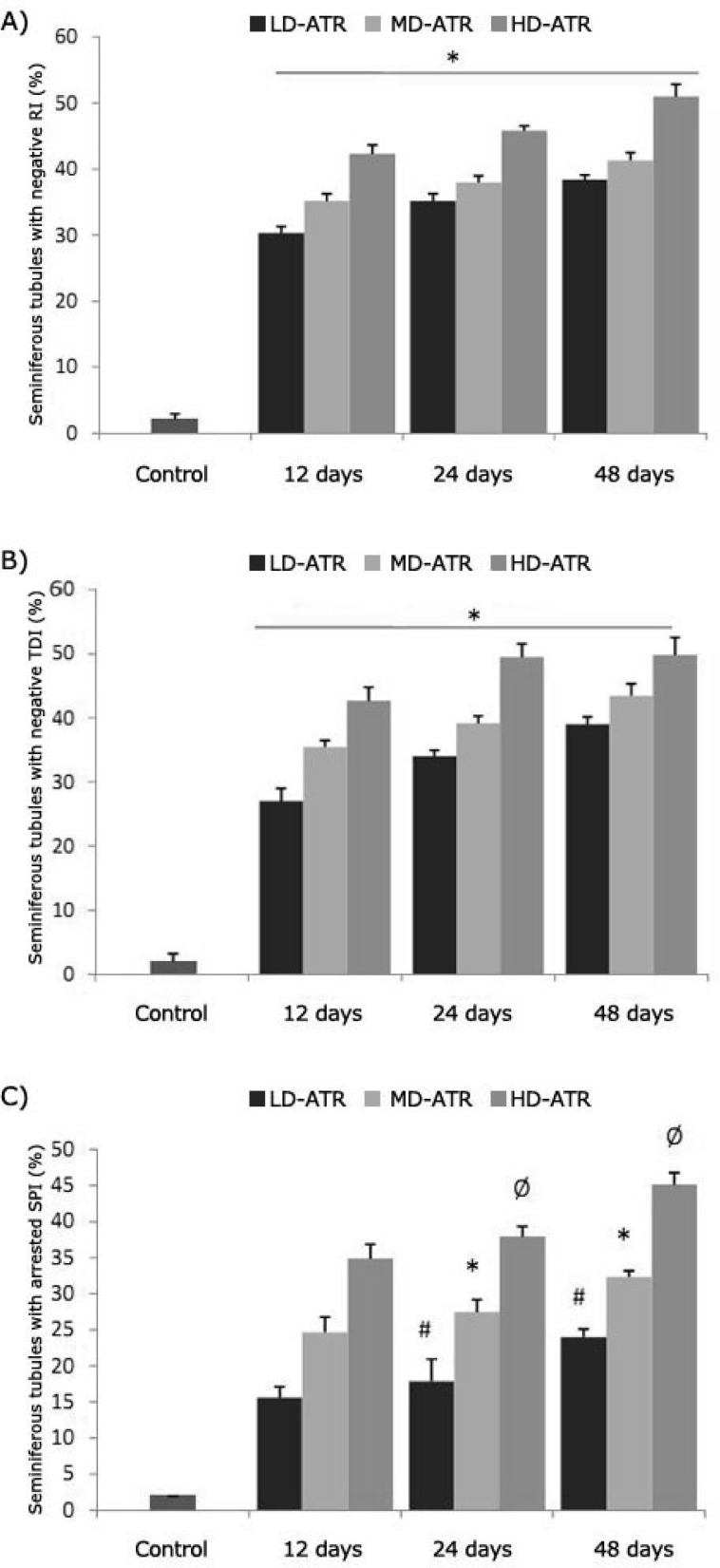
Percentage of the seminiferous tubules with negative RI (A), TDI (B) and SPI (C); stars in figures A and B are indicating significant differences between test groups with each other and with control group. In figure C, #, *, Ø are indicating significant differences (P ≤ 0.05) and there are significant differences between all test groups and control group (P ≤ 0.05). All data are presented as mean ± SD.

**Atrazine induced detrimental effects on testicular tissue. **Observations demonstrated that the testes in the test groups showed a remarkable atrophy associated with severe edema in the interstitial connective tissue. Majority of the interstitial blood vessels were revealed with vasodilatation and thrombosis. Histological examinations for Leydig cells showed that, in ATR-received animals the Leydig cells reduced in number per one mm^2 ^of the interstitial connective tissue. The Leydig cells were manifested with hypertrophic cytoplasms and gathered close to dilated vessels. In contrast, these cells were distributed in wide areas of interstitial tissue in control groups (Fig. 1). Light microscopic observations revealed that in ATR-exposed animals the percentage of STs with dissociated germinal cells increased dose- and time-dependently. Accordingly, the HD-ART-administered groups were exhibited significantly (*P* < 0.05) a higher disruption in germinal epithelium. No considerable cell dissociation was observed in the control group. The height of the germinal epithelium (GE) decreased in test animals. The STs in HD-ART-administered animals showed negative tubular differentiation (TDI), and repopulation indexes (RI). Up to 30% of the STs in HD-ART group revealed the negative spermiogenesis index (SPI). The data for the percentage of STs with negative RI, TDI and SPI are presented in Figure 2. After 48 days the giant cells were observed in 21.60 ± 2.07% of the STs in HD-ART-administered animals. No giant cells were observed in other the test and control animals.

**Table 1:** Average of body weight gain (BWG) and testicular weight gain (TWG) in different test and control-sham groups. All data are presented as mean ± SD.

**Groups**	**B.W.G**			**T.W.G**		
	**12**	**24**	**48**	**12**	**24**	**48**
**Control-sham**	215.00 ± 5.77	**-**	**-**	66.00 ± 1.00	**-**	**-**
**LD-ATR**	208.20 ± 0.56	198.70 ± 1.20	194.20 ± 2.77	61.40 ± 1.14	59.00 ± 1.00	54.20 ± 1.09
**MD-ATR**	193.60 ± 1.34	190.80 ± 0.83	189.20 ± 0.83	51.60 ± 1.14	49.00 ± 1.58	47.80 ± 0.83
**HD-ATR**	180.60 ± 2.70	174.80 ± 2.58	170.60 ± 1.94	43.85 ± 0.75	41.33 ± 1.21	40.33 ± 1.03

There are no significant differences between groups (*P* > 0.05).

**Atrazine reduced the cytoplasmic carbohydrate and elevated unsaturated fatty acids in spermatogenesis cells series. **Special staining for CH showed that in ATR-received animals the ratio of cytoplasmic CH increased in the first three layers of the STs. After 48 days the animals in HD-ART group revealed unstained spermatogonia and spermatocyte type I cells in more than 20% of the STs. In contrast, the first three layers of the germinal epithelium in the control rats manifested a dense reaction sites for PAS staining. Light microscopic studies for Sertoli cells (SCs) cytoplasmic CH ratio showed that in ATR-exposed groups, the number of CH negative SCs per one ST increased by the time and dose. 

**Fig. 3:**
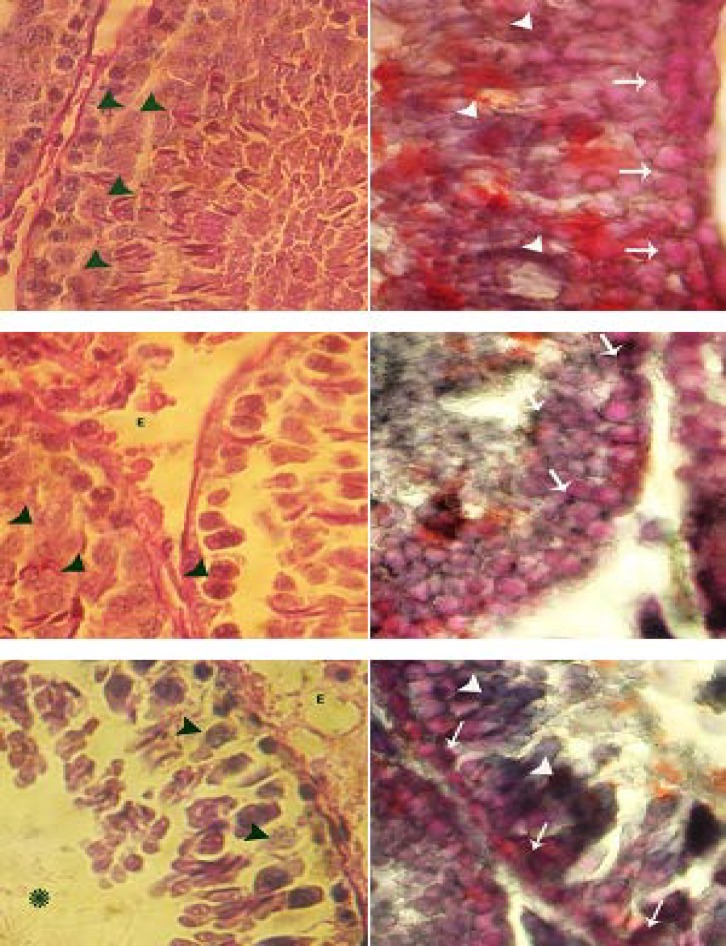
Cross sections from testes; histochemical staining for CH (left column) and UFA (right column). Control group (first row); note the PAS positive spermatogonia and spermatocyte type I cells (black head arrows), the SB-B negative cells spermatogonia and spermatocyte type I cells (white arrows) and densely SB-B stained spermatids (white head arrows). LD-ATR-induced group (mid row); note the edema in the interstitial connective tissue (E) and faint PAS stained spermatocyte type I cells (black head arrows) and faint SB-B stained spermatogonia and spermatocyte type I cells (white arrows). High dose ATR-induced group (last row); note the PAS negative spermatocyte type I cells (black head arrows) with severe edema in the connective tissue (E). The spermatogonia and type I spermatocytes are presented with dark SB-B stained cytoplasms (white arrows) and the spermatids are densely SB-B stained (white head arrows). Periodic acid shift (right column) and Sudan Black-B staining (left column), 400×.

Special staining for cytoplasmic UFA illustrated that despite to the control animals in ATR- administered groups the cytoplasmic ratio of the UFA increased in all first three layers of the GE. The spermatogonia and spermatocyte type I cells in particular manifested a dense lipidophilic cytoplasm. On the other hand, in the test animals the UFA positive SCs were increased in number per one ST in comparison to the control animals. The spermatids (spherical and elongated cells) were observed with dense UFA positive foci in all groups. While the animals in 48 days HD-ART group showed lower reaction sites for lipid staining (Fig. 3). 

**Atrazine lowered the serum levels of testosterone, INB, FSH and LH. **Biochemical analyses showed that the serum level of testosterone, FSH, LH and INB significantly (*P *≤ 0.05) decreased depending on dose and time. Accordingly the animals in 48 days HD-ART-administered group were manifested with the lowest level of the mentioned hormones (Fig. 4). 

**Fig 5:**
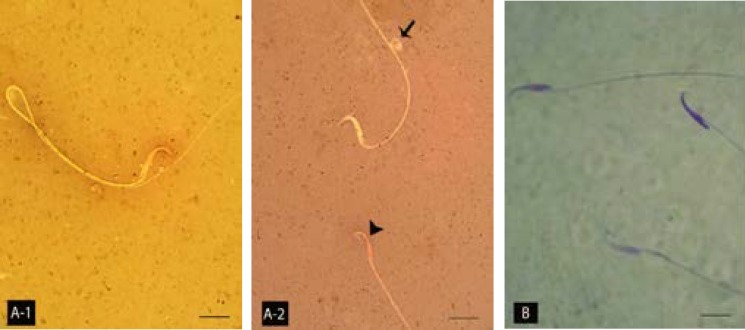
Light microscopic architecture of sperms; (A-1) normal sperm with unstained cytoplasm and (A-2) morphologically abnormal sperm with cytoplasmic droplet on the tail piece (arrow) and the dead sperm with stained cytoplasm (Head arrow). (B) Two nuclear immature sperms with faint stained nucleuses and one nuclear matured sperm on top right hand side are showed. A-1 and A-2, Eosin-negrosin and B, Aniline-blue staining, 1000×.

**Fig. 6:**
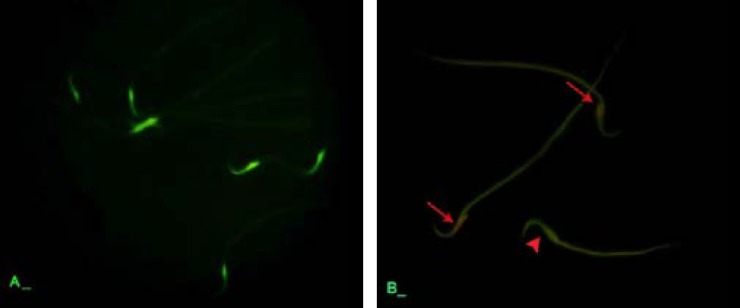
Epi-fluorescent architecture of sperms; (A) control group, note the sperms with light green florescent stained nucleus indicating normal DNA integrity. (B) ATR-induced group, the sperms with damaged DNA are presented with orange florescent (arrows) stained nucleuses and the normal sperm with light green (head arrow). Acridine orange staining, 1000×.

**Atrazine elevated the sperm abnormality, DNA disintegrity and nuclear immaturity. **Observations demonstrated that the sperm count decreased in ATR-received animals and it substantiated by the time and depending on dose. Accordingly, the HD-ART-administered animals revealed a significant (*P* ≤ 0.05) sperm content reduction in comparison to other the test and control animals. Eosin-negrosin staining for sperm mortality and morphological abnormalities showed that the percentage MI sperms increased in ATR-induced animals. This impairment advanced by the time in all test groups. Aniline blue staining for sperms protamine-packed-DNA showed that majority of the sperms (more than 80%) in 48 days HD-ART-administered were nuclear immature. The protamine expression was decreased in the spermatid’s early maturation process (Fig. 5). Further analyses for sperm DNA integrity showed the percentage of sperms with damaged DNA increased by the time in all ATR-administered groups. Comparing of the DNA damage between the test groups showed that the rate of DNA disintegrity significantly (*P* < 0.05) increased in HD-ART-administered animals which received ATR for 48 days (Fig. 6). The data for sperm parameters are presented in Table 2.

**Table 2:** Average of sperm count, nuclear and morphological immaturity and sperm DNA damage in different test and control-sham groups; all data are presented in mean ± SD

**Groups**	**Nuclear Immature Sperms (%)**			**Morphological immature sperms (%)**		
	**12**	**24**	**48**	**12**	**24**	**48**
**Control**	10.20 ± 1.48	-	-	9.83 ± 1.47	-	-
**LD-ATR**	67.80 ± 2.16	72.80 ± 1.92	77.00 ± 1.41	69.16 ± 1.16	71.34 ± 1.75	73.66 ± 1.75
**MD-ATR**	71.00 ± 1.41	76.80 ± 1.30	81.80 ± 1.78	71.50 ± 1.37	74.50 ± 1.64	77.08 ± 1.21
**HD-ATR**	74.60 ± 1.14	80.00 ± 1.22	83.40 ± 0.89	73.83 ± 1.60	77.33 ± 1.50	83.00 ± 2.25
	**Sperm Count (×10** ^6^ **)**			**Sperm DNA disintegrity (%)**		
**Control**	74.83 ± 1.16	-	-	10.83 ± 0.75	-	-
**LD-ATR**	65.15 ± 0.75	60.16 ± 1.47	55.16 ± 1.94	52.66 ± 2.16	57.16 ± 1.72	64.00 ± 1.09
**MD-ATR**	57.17 ± 1.72	52.50 ± 1.04	50.83 ± 0.98	56.83 ± 0.75	60.84 ± 2.04	67.33 ± 1.03
**HD-ATR**	41.33 ± 1.21	30.45 ± 1.54	26.50 ± 2.16	66.34 ± 1.50	68.50 ± 0.83	73.16 ± 0.72

**Fig . 4:**
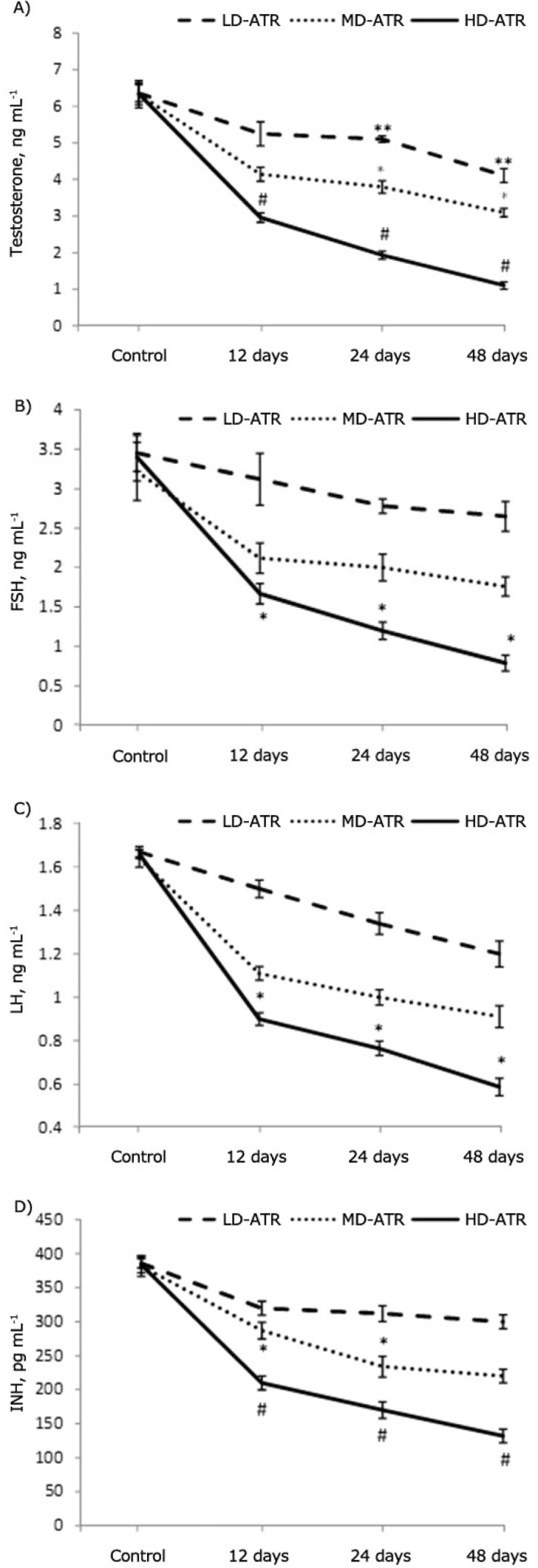
Serum levels of testosterone **(A)**, FSH **(B)**, LH **(C)** and INH **(D)**; *, ** and # are presenting significant differences (*P* ≤ 0.05) between marked groups. There are significant differences (*P* ≤ 0.05) between all test groups and control animals. All data are presented as mean ± SD.

**Fig. 7:**
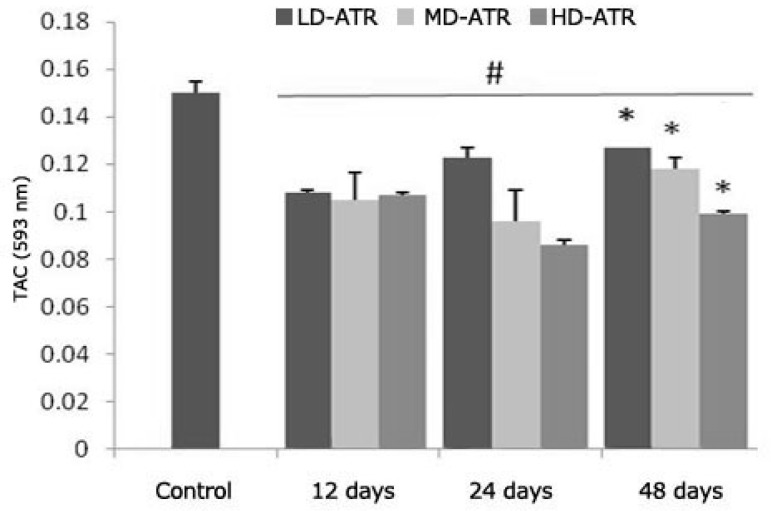
Average of TAC in different test and contol groups, * is indicating significant differences between marked groups and # is presenting significant differences between all test groups and control animals. All data are presented as mean ± SD

**Atrazine lowered serum TAC. **Biochemical analyses showed that in the ATR-exposed groups, the serum TAC decreased by the time and depending on dose. The animals that received the high dose of ATR for 48 days showed the lowest antioxidant power (0.10 ± 0.005) in comparison to the other test and control groups (Fig. 7).

## Discussion

The main purpose of the current study was to investigate the detrimental effects of ATR on male reproductive system. Our results showed that ATR influences the Leydig cells, SCs and GE healthiness by interfering with gonadotrophic and testicular endocrine function. Moreover, ATR administration could affect the GE cytoplasmic CH and UFA ratio, too. At the same time the current study illustrated that ATR can disturb the total antioxidant capacity, which may affect the sperms nuclear maturity and DNA integrity.

According to previous reports the pituitary gland is considered as a target tissue for ATR and in male offspring this compound is capable to induce gonadal malformation and/or immaturity.^12^^,^^16^^,^^17^ Our biochemical analyses revealed that a statistically significant reduction in FSH and LH levels (more than 85% reduction in serum levels) was occurred in HD-ART-administered group, while no statistically significant differences was observed in LD and MD-ATR-administered groups. Thus our results confirmed the previous reports and extended the fact that the ATR-induced detrimental impacts are in a dose- and time-dependent manner. The spermatogenesis and cellular integrity in mammals depend largely on testosterone production by Leydig cells in response to stimulation by FSH and LH.^16^^-^^19^ The biochemical analyses in this study revealed that after significant reduction in serum levels of FSH and LH, the serum level of the testosterone decreased in ATR animals. On the other hand histological analyses for Leydig cells distribution showed that these cells manifested with a remarkable degeneration which was characterized by decreased number of these cells per one mm^2 ^of the interstitial connective tissue. FSH stimulates the SC to synthesis an androgen binding protein required to keep the high concentrations of testosterone.^18^^,^^21^ Moreover it is well understood that any reduction in testosterone level can lead to SCs malfunction and also a severe reduction in INH-B synthesis (imidoclopride). Therefore a significant reduction in serum level of testosterone which is found in this study may explain the malfunctioning of SCs in ATR-exposed animals. To show the effect of ATR on SCs, the SB-B staining as a biomarker of phagocytosis and PAS staining technique for diagnosis of degenerated cells were used.^15^^,^^22^ In parallel, the biochemical findings confirmed the histological observations as the serum level of INH-B decreased time- and dose-dependently in ATR-exposed animals. Thus it could be concluded that in addition to pituitary hormones, ATR can affect the testicular endocrine functions by degenerating two important Leydig and SCs (cells participating in testicular endocrine functions). SCs are known as cells that guarantee the germinal epithelium (GE) integrity.^6^^,^^23^ Therefore any SCs degeneration can influence the GE integrity. Our histological analyses lightened this theory and showed that the majority of the STs were undergone to GE dissociation 24 and 48 days after exposing to medium and high dose levels of ATR. Therefore, it would be suggestible that ATR is able to influence the spermatogenesis process by inducing detrimental effects on gonadal endocrine functions and consequently stopping the spermatogenesis process during long term exposure. This suggestion is supported by increased negative TDI and RI in more than 49.85 ± 2.78 and 49.50 ± 1.87 percent of the STs, respectively. 

To clarify the exact effect of ATR on germinal epitheliums CH and UFA metabolism and also to identify the cytoplasmic ratio of the CH and UFA foci the SB-B and PAS staining techniques were used. It is well proofed that the three first layers of the GE (spermatogenesis processes cell series) are strongly participating in mitosis and meiosis divisions by using glucose as a main source of the energy.^24^ As the second three layers (spermiogenesis cell series, mainly spermatids) does not participate in cellular divisions, thus in normal conditions these cells use UFAs and SCs inter-cytoplasmic glucose as a main energy sources to continue their biological activities.^15^ Our histochemical observations revealed that following ATR exposure the spermatogenesis cell colonies showed faint PAS reaction sites. In contrast, these cells were presented with dense cytoplasmic UFA accumulation. This impairment developed by the time especially in animals that received the high dose of ATR. Hence it could be concluded that after ATR exposure the glucose metabolism and/or transportation through lamina properia are disrupted and results in switch of energy source from glucose to UFAs in the spermatogenesis cells series. Consequently these cells lost their biological activities. The arrested spermatogenesis and negative TDI plus densely SB-B stained spermatogonia and spermatocyte type I cells approved this theory. 

According to previous reports a variety of constituents in male genital system are intended to generate ROS, including morphologically abnormal spermatozoa, antecedent germ cells degeneration, infiltrated leukocytes in testes and semen.^15^ Our findings illustrated that in ATR-administered animals the possible role of imbalanced oxidative stress which has been shown by TAC measurement, resulted in an elevated death and MI sperms plus degenerated germinal cells. The results of TAC determination confirmed that TAC decreased in all ATR groups in a dose- and time dependent manner. According to previous studies the sperm DNA disintegrity and sperms morphological maturation positively correlated with amount of pathologically formed ROS and negatively associated with the TAC.^14^^,^^16^ Other studies have also suggested that the presence of spermatozoa with damaged DNA may be the result of an impaired chromatin packing.^25^ Our light microscopic examinations for sperm morphological maturation and DNA integrity showed that after 24 and 48 days the percentage of the morphologically immature sperms and sperms with damaged DNA increased in ATR-induced animals. Furthermore, the aniline-blue staining showed that the percentage of the sperms with immature nucleus increased in time- and dose-depending manner. Thus we can conclude that ATR exerts its impacts not only by increasing the DNA disintegrity but also by affecting DNA packing process. The DNA packing is attributed to the rate of protamine expression during early maturation phase.^26^ Thus ATR influences the maturation processes and/or protamine expression therefore degenerates the sperm DNA packing. This hypothesis supported by a lowered percentage of sperms with positive aniline-blue stained nucleus. Referring to earlier studies the increased formation of ROS has been correlated with the reduction of sperm motility.^27^^,^^28^ The link between ROS and reduced motility can be explained by two hypothesis; a waves of events that result in an intensive decrease in axonemal protein phosphorylation and sperm immobilization and secondly, free radicals such as H_2_O_2_ can diffuse across the membranes into the cells and inhibit the activity of enzymes such as G6PDH.^29^^,^^30^ Our observations demonstrated that after 48 days the ATR-induced rats manifested with high percentage of immotile sperms. It may be occurred due to the fact that spermatozoa are particularly susceptible to oxidative stress because their plasma membranes are so enriched with unsaturated fatty acids. In order to exactly identify the rate of sperm plasma membrane damage, we used eosin-negrosin staining technique and it confirmed this hypothesis by illustrating increased number of the death sperms with defected plasma membrane. Hence, our findings support the hypothesis that ATR-induced damages on sperms associates with increased oxidative agents in long time and induces immobilization of the sperms by causing severe damage in plasma membrane. 

According to our results ATR exerts its effects primarily via the pituitary-testes axis by decreasing the synthesis of FSH and LH. In turn this impairment leads to Leydig and SCs dysfunction respectively. Consequently the remarkable alteration in cytoplasmic CH and UFA in spermatogenesis cells exhibits, which attributes to SCs malfunction. Ultimately, the ATR-induced oxidative stress accompanies with inadequate energy supplement results in sperm DNA disintegrity and abnormal DNA packing. 
